# From molecular subgroups to molecular targeted therapy in rheumatoid arthritis: A bioinformatics approach

**DOI:** 10.1016/j.heliyon.2024.e35774

**Published:** 2024-08-06

**Authors:** Yangyang Xu, Zhenyu Yang, Tengyan Wang, Liqiong Hu, Songsong Jiao, Jiangfei Zhou, Tianming Dai, Zhencheng Feng, Siming Li, Qinqqi Meng

**Affiliations:** a Guizhou Medical University, Guiyang City, Guizhou Province, China; b Jinan University, Guangzhou, Guangdong Province, China; c Guangzhou Red Cross Hospital Affiliated of Jinan University, Guangzhou, Guangdong Province, China; d Xuzhou New Health Hospital, North Hospital of Xuzhou Cancer Hospital, Xuzhou City, Jiangsu Province, China; e Guizhou Hospital of The First Affiliated Hospital, Sun Yat-Sen University, Guiyang City, Guizhou Province, China

**Keywords:** Rheumatoid arthritis, Heterogeneity, Molecular subgroup, Transcriptomics, Diagnosis

## Abstract

**(1)Background:**

Rheumatoid Arthritis (RA) is a heterogeneous autoimmune disease with multiple unidentified pathogenic factors. The inconsistency between molecular subgroups poses challenges for early diagnosis and personalized treatment strategies. In this study, we aimed to accurately distinguish RA patients at the transcriptome level using bioinformatics methods.

**(2)Methods:**

We collected a total of 362 transcriptome datasets from RA patients in three independent samples from the GEO database. Consensus clustering was performed to identify molecular subgroups, and clinical features were assessed. Differential analysis was employed to annotate the biological functions of specifically upregulated genes between subgroups.

**(3)Results:**

Based on consensus clustering of RA samples, we identified three robust molecular subgroups, with Subgroup III representing the high-risk subgroup and Subgroup II exhibiting a milder phenotype, possibly associated with relatively higher levels of autophagic ability. Subgroup I showed biological functions mainly related to viral infections, cellular metabolism, protein synthesis, and inflammatory responses. Subgroup II involved autophagy of mitochondria and organelles, protein localization, and organelle disassembly pathways, suggesting heterogeneity in the autophagy process of mitochondria that may play a protective role in inflammatory diseases. Subgroup III represented a high-risk subgroup with pathological processes including abnormal amyloid precursor protein activation, promotion of inflammatory response, and cell proliferation.

**(4)Conclusion:**

The classification of the RA dataset revealed pathological heterogeneity among different subgroups, providing new insights and a basis for understanding the molecular mechanisms of RA, identifying potential therapeutic targets, and developing personalized treatment approaches.

## 1 Introduction

Rheumatoid Arthritis (RA) is a prevalent autoimmune disease primarily affecting the joints, leading to arthritis and joint functional impairment [1]. According to the Global Burden of Disease study in 2017 [2], the global incidence of RA is around 0.27 %, with females experiencing a 2–3 times higher incidence than males [[3], [4], [5], [6]]. The age-standardized global prevalence of RA has increased by 7.4 %, and the incidence has risen by 8.2 %. RA has become a significant public health challenge worldwide.

The exact etiology of RA remains unknown, but it is believed to be associated with genetics, immune infiltration, infections, chronic inflammation, and viral infections (including *Staphylococcus aureus* and enteroviruses) [2,[7], [8], [9]]. Due to the complex and diverse pathogenesis, the diagnosis and treatment of RA have been a considerable challenge. Early diagnosis is crucial for implementing effective therapeutic measures as early intervention significantly improves patient outcomes [10]. However, identifying individuals at risk of RA and making accurate diagnoses in the preclinical stage have been problematic. Currently, RA diagnosis relies mainly on medical history, clinical manifestations, and a high index of suspicion [11]. Nevertheless, in the early stages of the disease, symptoms may be atypical, or serum rheumatoid factor (RF) or anti-cyclic citrullinated peptide (anti-CCP) tests may be negative, leading to delayed diagnosis [12].

To achieve more accurate RA diagnosis and understand its pathological process, exploring RA diagnostic biomarkers as adjunct tools for the pathological process of RA is vital for personalized treatment. Some biomarkers, such as RF and anti-cyclic citrullinated peptide antibodies (ACPA), have been extensively studied and demonstrated certain value in RA diagnosis [13,14]. Additionally, specific gene expression profiles (e.g., SAA4, RBP4, VDBP) [15] and molecular features (secretory pathways and interleukin receptors) [16] have been proposed to be associated with RA subgroups and disease progression.

In recent years, the development of high-throughput genomic sequencing has provided new opportunities for biomarker research in RA. By systematically identifying co-expressed gene modules associated with RA development and determining gene loci related to RA susceptibility, we can gain a better understanding of the pathological mechanisms and biological heterogeneity of RA. A study discovered 87 highly correlated and diagnostically valuable genes, including FADD, CXCL2, and CXCL8, in co-expression modules associated with RA [17]. Current similar biomarker studies mostly focus on differential expression between RA and non-RA samples, without effectively distinguishing the severity of pathological markers. This research is based on tumor samples and utilizes gene expression patterns to differentiate subgroups, revealing heterogeneity among tumors, guiding treatment, and predicting clinical outcomes [18]. By segregating RA cases into different subgroups, each subgroup may exhibit distinct functions and clinical characteristics.

Through in-depth exploration of molecular subgroups in RA, we can discover more precise biomarkers, facilitating transcriptome-based specific targeting for early diagnosis and treatment. The core genes in these subgroups may hold promising diagnostic value and could potentially provide new leads for developing novel personalized treatment strategies.

## 2 Materials and methods

### 2.1 Experimental design

.

### 2.2 Data acquisition

**Fig. 1:**
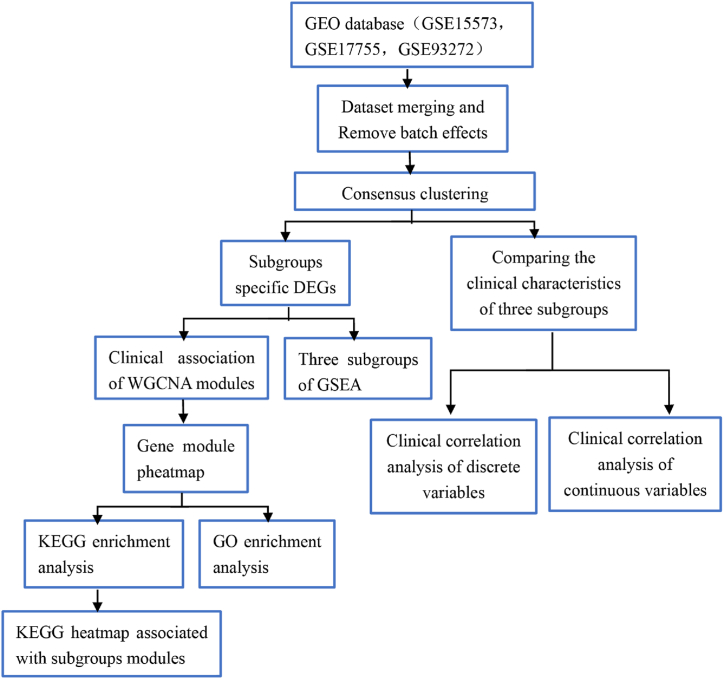
Illustrates the experimental design.

**Fig. 2:**
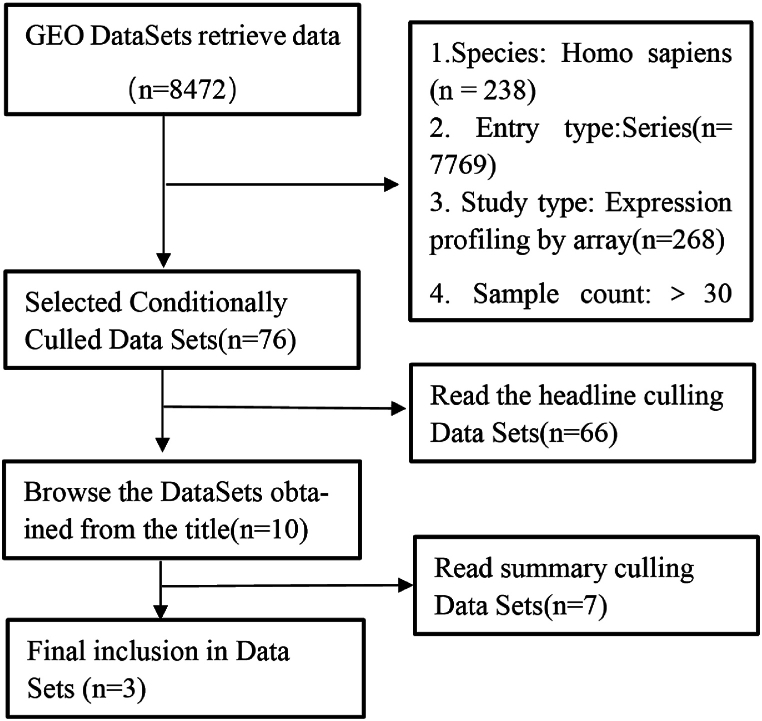
Prisma flowchart for dataset filtering.

Rheumatoid Arthritis (RA) gene expression profiling data were obtained from the GEO database (https://www.ncbi.nlm.nih.gov/geo/) (see Fig. 1). Relevant gene expression datasets were identified by searching with keywords “RA” and “rheumatoid arthritis” while filtering for series and expression profiling by array. Non-human test samples were excluded. Datasets such as non-RA diseases, pharmacological interventions, etc. were then excluded by reading the headings, and datasets of non-peripheral blood origin were excluded by reading the abstracts(Fig. 2). Three datasets were finally selected, with the following accession numbers: GSE15573 (Teixeira et al., 2009; platform probe: Illumina human-6 v2.0 expression beadchip), GSE17755 (Lee et al., 2010; platform probe: Hitachisoft AceGene Human Oligo Chip 30K 1 Chip Version), and GSE93272 (Tasaki et al., 2018; platform probe: [HG-U133_Plus_2] Affymetrix Human Genome U133 Plus 2.0 Array).

### 2.3 Batch effect removal and Principal Component Analysis (PCA)

The datasets were merged, and duplicate data points were averaged. The data was log2-transformed and normalized. Functional correction batch effects was performed by estimating the mean and variance of each batch independently using each gene. PCA plots before and after normalization were generated using the orthogonal transformed PCA method.

### 2.4 Consensus clustering analysis of gene expression data

Consensus clustering was performed using the k-means algorithm with Spearman correlation as the distance metric. The maximum number of clusters (maxK) was set to 10, and consensus clustering scores greater than 0.8 were used to select the optimal clustering with 3 clusters.

### 2.5 Protein interaction network (PPI) and network core genes (cytoHubba)

The Protein-Protein Interaction (PPI) analysis was conducted using the online tool available at https://cn.string-db.org/. Gene data extracted from subpopulation clustering results were selected for *Homo sapiens* species. The network type utilized was the full STRING network, where the meaning of network edges was based on evidence. A minimum required interaction score of medium confidence (0.400) was set, and network display options were adjusted to hide disconnected nodes. The results were exported in tab-separated values (TSV) format. Subsequently, the TSV file was imported into cytoHubba software, with node1 designated as the Source Node and node2 as the Target Node. Nodes' scores were calculated to obtain the gene eigenvalue score. Finally, the number of core genes was set to 10, and the core gene network graph was generated, output, and saved for further analysis.

### 2.6 Comparison of clinical features between subgroups (Categorical and continuous variables)

Clinical analysis features with female patients as discrete variables were selected based on the clustering results. Analyses were performed using the paired proportion test, and the results were visualized in bar charts. Additionally, pairwise comparisons were performed within the clustering results, and the Multi-group ANOVA test was used to test the statistical significance of continuous clinical features, including age, Crohn's Disease Activity Index (CDAI), C-reactive protein(CRP), erythrocyte sedimentation rate(ESR), Matrix metalloproteinase-3(MMP3)and Simplified Disease Activity Index(SDAI).

### 2.7 ANOVA analysis of subgroup clustering and clinical information

Clinical information extracted from the subgroup clustering results was subjected to Bartlett's test to assess variance homogeneity between SDAI and subgroups. Subsequently, ANOVA analysis using the ANOVA test was conducted to establish the correlation model between clinical features (Age, CDAI, CRP, ESR, and MMP3) and SDAI index, and the ANOVA results were obtained.

### 2.8 Analysis of subgroup-specific upregulated genes

Identification of subgroup-specific upregulated genes was achieved by comparing the cases in specific subgroups with those in each other subgroup. Wilcoxon's sum-rank test was used to detect differentially expressed genes, and Benjamini-Hochberg(Control false positive rate) correction was applied to adjust p-values, setting significance threshold at p < 0.05. For determining specific upregulated genes, we required an absolute mean difference >0.2. For each gene, the expression mean of the normal control group was subtracted from the expression mean of the specific subgroup to obtain the absolute value of the mean difference.

### 2.9 Gene set enrichment analysis (GSEA)

GSEA was performed using version 4.3.2 of GSEA software. GSEAPreranked mode was selected for gene set enrichment analysis, comparing rheumatoid arthritis and normal control groups for each subgroup based on Studentʼs *t*-test P-values sorted.

### 2.10 Weighted gene co-expression network analysis (WGCNA)

Expression data of specific genes were extracted for sample clustering and detection of outlier samples. The appropriate power range (1:20) for network construction was determined by power calculations. The topological overlap matrix (TOM) of gene-to-gene connections was calculated. Based on TOM, gene clustering and dynamic tree cutting were performed to identify similar gene modules. Furthermore, the correlation between each module and clinical features was analyzed, and heatmaps were generated.

### 2.11 GO enrichment and KEGG pathway enrichment analysis

GO enrichment and KEGG pathway enrichment analysis were conducted for all modules. The enrichplot packages in R were used for gene function enrichment analysis with org.Hs.eg.db database and p-value and q-value filtering thresholds were set. Bubble plots were generated based on enrichment analysis results, and KEGG pathway heatmaps were produced according to the pathway analysis.

## 3 Results

### 3.1 Characteristics of the dataset

We downloaded a total of 465 peripheral blood samples from three independent studies from the GEO database, including 362 patients with rheumatoid arthritis (RA) and 103 healthy control individuals. Among these, the GSE93272 dataset provided relevant clinical indicators such as Age, Gender, SDAI, CDAI, CRP, ESR and MMP3.

### 3.2 Batch effect removal

**Fig. 3:**
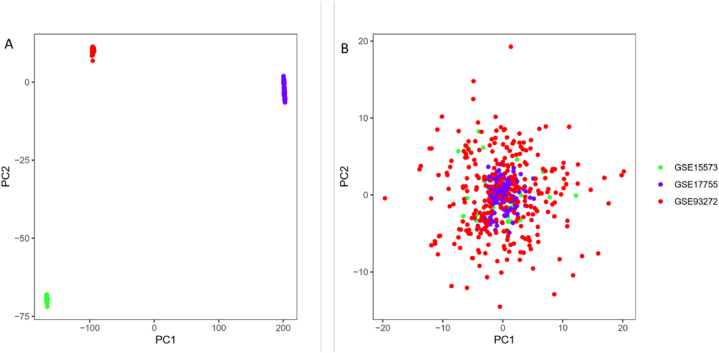
Principal Component Analysis (PCA) scatter plots, where each color represents different datasets. (A) Visualization of samples before batch effect removal. (B) Visualization of samples after batch effect normalization.

After merging the gene data from different datasets, we obtained 2019 genes. Principal Component Analysis (PCA) plots were visualized in two-dimensional space before and after normalization using R. The PCA plot before batch effect removal (Fig. 3A) showed separation of datasets, indicating significant differences between them. In contrast, the normalized PCA plot (Fig. 3B) showed a random distribution, suggesting successful batch effect removal.

### 3.3 Clustering of rheumatoid arthritis subgroups

**Fig. 4:**
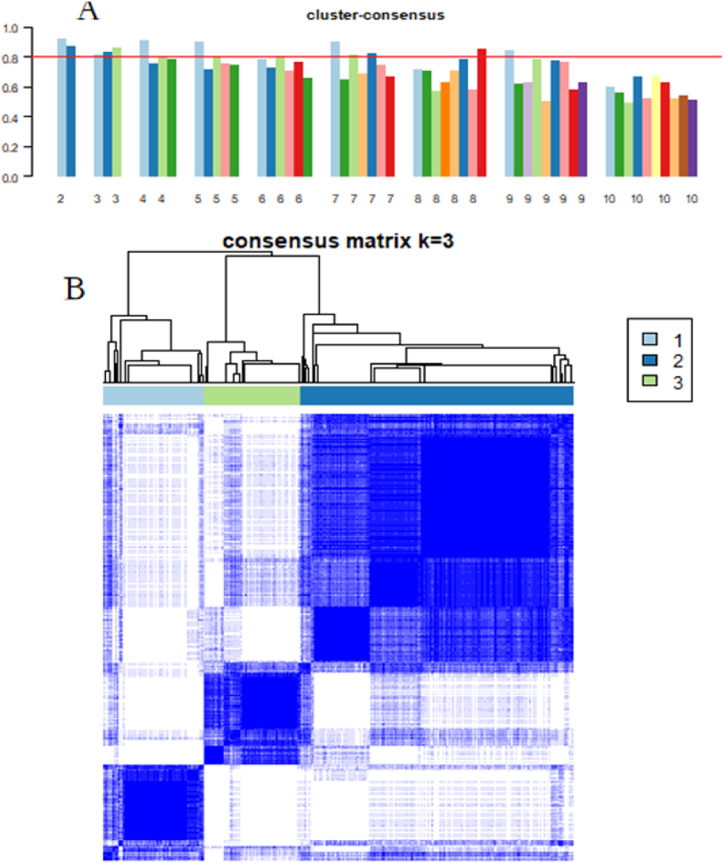
Consensus clustering analysis of RA samples. (A) Bar plot of consensus scores for clustering from k = 2 to k = 10, showing that three subgroups have higher consensus scores (>0.8). (B) Consensus matrix for clustering with k = 3.

**Fig. 5:**
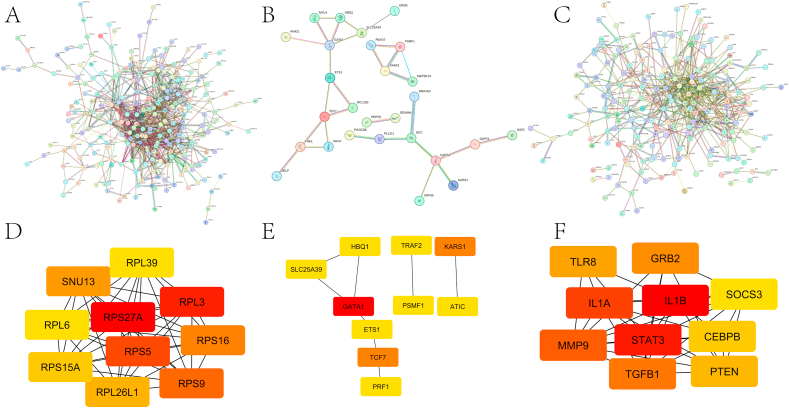
Core genes of subgroups. (A) PPI interactions network of subgroup I. (B) PPI interactions network of subgroup II. (C) PPI interactions network of subgroup III. (D) Hub genes of subpopulation I. (E) Hub genes of subpopulation II.(F) Hub genes of subpopulation III.

We performed clustering analysis on the gene expression profiles of 362 rheumatoid arthritis samples after batch effect removal, dividing them into 2 to 10 subgroups. The results indicated that two subgroups and three subgroups had consensus scores above 0.8 (Fig. 4A). Considering that more detailed analysis could be achieved with richer subgroups, we finally selected three subgroups as the most robust classification. Based on the consensus matrix, there were 78, 210, and 74 samples with highly similar gene expression patterns in subgroups I, II, and III, respectively, with significant differences in expression patterns among them (Fig. 4B) (see Fig. 5).

### 3.4 PPI network construction and identification of core genes

After removing disconnected nodes, a Protein-Protein Interaction (PPI) network was constructed for the three subgroups (Figure A, B, C) using the STRING tool. Subsequently, the CytoHubba plugin was employed to identify highly connected genes (Hub genes) within this network. The results revealed that in subcluster I, the most highly connected Hub genes were RPS27A, RPL3, RPS5, RPS9, RPS16, SNU13, RPL26L1, RPS15A, RPL6, and RPL39. In subcluster II, the most highly connected Hub genes included GATA1, TCF7, KARS1, PRF1, HBQ1, PSMF1, TRAF2, SLC25A39, ETS1, and ATIC. Similarly, subcluster III exhibited Hub genes with the highest connectivity, namely IL1B, STAT3, IL1A, MMP9, TGFB1, GRB2, TLR8, PTEN, CEBPB, and SOCS3 (Fig. D, E, F).

### 3.5 Clinical features of RA subgroups

**Fig. 6:**
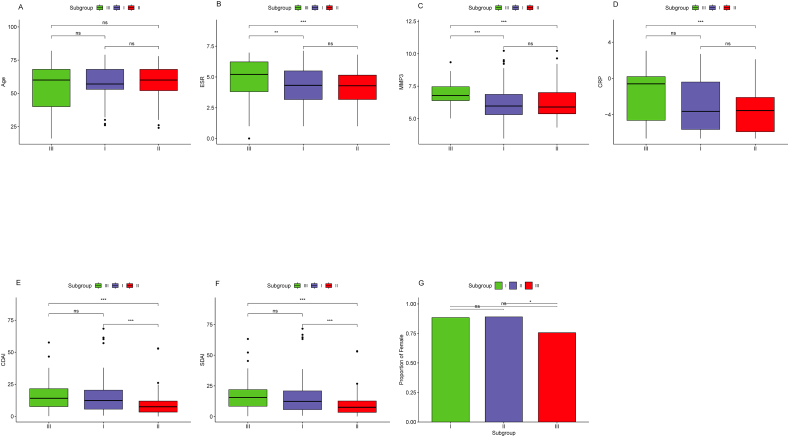
Clinical features of subgroups. (A) Comparison of age among subgroups. (B) Comparison of ESR among subgroups. (C) Comparison of MMP3 among subgroups. (D) Comparison of CRP among subgroups. (E) Comparison of CDAI among subgroups. (F) Comparison of SDAI among subgroups. (G) Comparison of the proportion of females among subgroups. Pairwise *t*-test, nsP >0.05; *P < 0.05; **P < 0.01; ***P < 0.001.

Correlation analysis of Age (Fig. 6A) among different RA subgroups showed no statistically significant differences (P > 0.05). Subgroup III showed significantly higher levels of ESR, MMP3, CRP, CDAI, and SDAI compared to Subgroup II (Fig. 6B–F), with statistical significance (P < 0.001). Additionally, the proportion of females in Subgroup II was higher than that in Subgroup III (P < 0.05) (Fig. 6G). Furthermore, ESR and MMP3 were significantly higher in Subgroup III than in Subgroup I (P < 0.001 and P < 0.01, respectively) (Fig. 6B and C), while CRP, CDAI, SDAI, and the proportion of females showed no significant differences (P > 0.05) (Fig. 6D–F). Both CDAI and SDAI in Subgroup I were significantly higher than in Subgroup II (P < 0.001) (Fig. 6E and F), while ESR, MMP3, CRP, and the proportion of females showed no significant differences (P > 0.05) (Fig. 6B–D). Subgroup III showed a higher proportion of male patients (Fig. 4G) and significantly higher levels of ESR, MMP3, CRP, CDAI, and SDAI (Fig. 6B–F), indicating a more severe disease condition. Subgroup II had the lowest levels of ESR, MMP3, CRP, CDAI, and SDAI (Fig. 6B–F), suggesting the mildest disease condition.

### 3.6 ANOVA analysis

The ANOVA analysis results for Age, Clinical Disease Activity Index (CDAI), C-reactive protein (CRP), Erythrocyte Sedimentation Rate (ESR), and MMP3 in relation to SDAI index are presented in Table 1. For each variable, the degrees of freedom (Df), total sum of squares (Sum Sq), mean square (Mean Sq), F-value (F value), and significance level (Pr(>F)) are reported. The results show that CDAI, CRP, ESR, and MMP3 have highly significant correlations with SDAI index (F-values: 27,246.516, 120.524, 49.704, and 57.209, respectively, P-values: 2e-16, 2e-16, 2.15e-11, and 9.79e-13), while Age has no significant correlation with SDAI index (F-value: 2.66, p-value: 0.104). Additionally, the interaction between CDAI, CRP, ESR, MMP3, and Subgroups is also significantly different (F-values: 8.316, 7.028, 3.227, and 8.737; P-values: 0.000327, 0.00109, 0.0415, and 0.000221, respectively). Overall, the ANOVA results indicate that Subgroup, CDAI, CRP, ESR, and MMP3, as well as the interaction between Subgroup, CDAI, CRP, ESR, and MMP3 with the SDAI index, have significant effects on the response variable. Furthermore, the small residual errors suggest a good fit of the model to the data.

**Table 1:** ANOVA analysis for models related to clinical features of subgroups.

	**Df**	**Sum Sq**	**Mean Sq**	**F value**	**Pr(** > **F)**
Subgroup	2	3488	1744.1	10.923	2.96e-05^c^
Age	1	425	424.7	2.66	0.104
Subgroup:Age	2	309	154.5	0.967	0.382
Residuals	226	36086	159.7		
Subgroup	2	3488	1744	1302.098	<2e-16^c^
CDAI	1	36495	36495	27246.516	<2e-16^c^
Subgroup:CDAI	2	22	11	8.316	0.000327^c^
Residuals	226	303	1		
Subgroup	2	3488	1744	17.08	1.24e-07^c^
CRP	1	12307	12307	120.524	<2e-16^c^
Subgroup:CRP	2	1435	718	7.028	0.00109^b^
Residuals	226	23077	102		
Subgroup	2	3488	1744	13.365	3.27e-06^c^
ESR	1	6486	6486	49.704	2.15e-11^c^
Subgroup:ESR	2	842	421	3.227	0.0415^a^
Residuals	226	29492	130		
Subgroup	2	3488	1744	14.243	1.49e-06^c^
MMP3	1	7005	7005	57.209	9.79e-13^c^
Subgroup:MMP3	2	2140	1070	8.737	0.000221^c^
Residuals	226	27674	122		

Note.

a P < 0.05.

b P < 0.01.

c P < 0.001.

### 3.7 Subgroup-specific upregulated genes and GSEA enrichment analysis

We set the mean filtering threshold to >0.2 and adjusted P-value to <0.05 as screening criteria to compare the gene expression profiles between different subgroups for differential expression analysis. A total of 604 subgroup-specific upregulated genes were identified, with 295, 58, and 251 genes specific to Subgroups I, II, and III, respectively (Supplementary File 1: subgroup-specific upregulated genes). Additionally, we analyzed the differentially expressed genes in each subgroup, finding 602, 141, and 567 genes specific to Subgroups I, II, and III, respectively. Compared to 119 differentially expressed genes between the normal and RA groups (Supplementary File 2: normal and RA groups differential genes), the number of differentially expressed genes increased significantly after subgroup separation, suggesting that subgroup analysis improves the detection of disease-related genes.

**Fig. 7:**
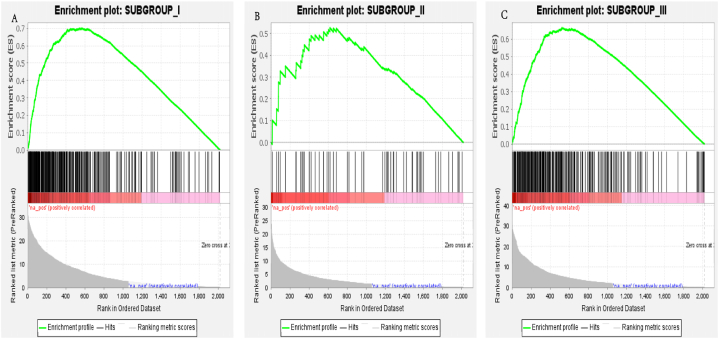
GSEA enrichment analysis results for subgroup-specific upregulated genes. (A) Significant upregulation of genes in Subgroup I (FDR <0.001), n = 295. (B) Non-significant upregulation of genes in Subgroup II (FDR >0.05), n = 58. (C) Significant upregulation of genes in Subgroup III (FDR <0.001), n = 251.

GSEA revealed that Subgroups I and III had significantly differentially upregulated genes compared to the control group (FDR <0.001, Fig. 7A–C), while no significant differences were observed for subgroup II-specific upregulated genes. This indicates that the subgroup-specific upregulated genes can effectively distinguish Subgroups I, III, and the control group.

### 3.8 WGCNA module analysis and subgroup-related heatmap

**Fig. 8:**
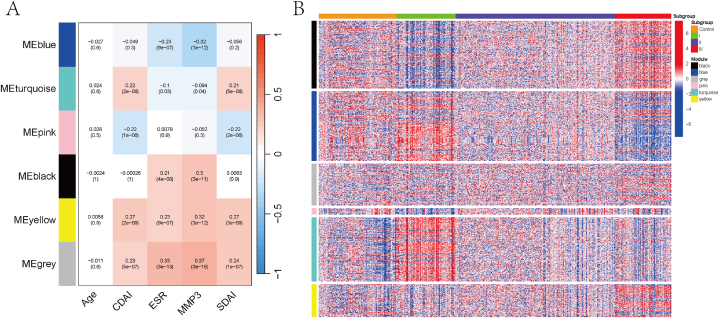
(A) WGCNA gene module analysis. (B) Correlation heatmap for module analysis. Note: P < 0.05 is considered statistically significant. Red indicates a positive correlation, and blue indicates a negative correlation.

**Fig. 9:**
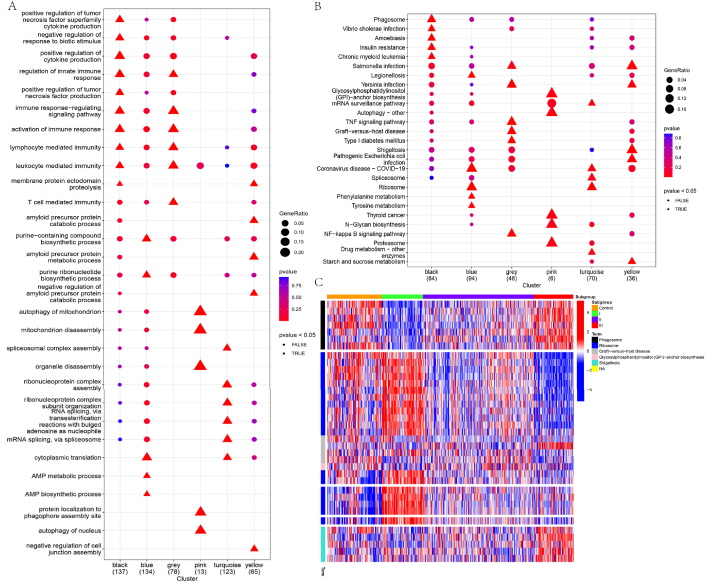
(A) GO enrichment analysis of module genes. (B) KEGG enrichment analysis of module genes. (C) Heatmap showing the correlation between KEGG enrichment results and module genes.

We conducted WGCNA module correlation analysis to explore the relationship of clinical features. Setting the minimum module size to 10, we calculated the correlation coefficients and corresponding P-values between Age, CDAI, ESR, MMP3, and SDAI with each module's specific upregulated genes (Fig. 8A). The module analysis results showed no significant correlation between Age and gene modules. The correlation heatmap generated from the module-gene output results (Fig. 8B) revealed that Subgroup I was positively correlated with the blue and turquoise modules, Subgroup II was positively correlated with the pink module, and Subgroup III was positively correlated with the black, grey, and yellow modules. These results suggest that subgroups cluster into different gene modules that are associated with clinical symptoms, indicating the stability of the subgroup clustering (see Fig. 9).

### 3.9 GO and KEGG enrichment analysis, and subgroup-related heatmap of KEGG enrichment results

GO enrichment analysis revealed that Subgroup I (genes from blue and turquoise modules) was mainly enriched in biological processes related to cytoplasmic translation, purine-containing compound biosynthesis, and AMP metabolism and biosynthesis pathways. These enriched pathways play crucial roles in cellular metabolism, protein synthesis, and regulation of gene expression. The cytoplasmic translation pathway involves genes such as DRG1, RBM4, RPL19, RPL22, RPL29, RPL3, RPL32, RPL6, RPS16, RPS21, RPS25, RPS27A, RPS5, and RPS9, which participate in ribosome assembly and protein synthesis processes. The purine-containing compound biosynthesis pathway includes genes such as ACSL4, ADSL, ATIC, ELOVL5, MTAP, NDUFAB1, NDUFB11, NT5E, PRPS2, and SLC25A12, which regulate purine metabolism within the cell. The AMP metabolism and biosynthesis pathway include genes such as ADSL, ATIC, and PRPS2, which are involved in AMP synthesis and metabolism. These results suggest that Subgroup I may play an important role in cellular metabolism and protein synthesis regulation.

Subgroup II (genes from the pink module) GO enrichment analysis showed that this subgroup is associated with autophagy, protein localization to the organelle, and organelle disassembly pathways. Autophagy is a cellular process of self-degradation that plays a critical role in cellular stress and metabolic balance. Genes such as ATG9A, FBXO7, and WDR45 in Subgroup II are involved in mitochondrial autophagy and organelle disassembly processes. Furthermore, the protein localization and organelle disassembly pathways are characteristic of Subgroup II and are essential for protein localization within the cell and maintaining organelle structure stability.

Subgroup III (genes from the black, grey, and yellow modules) GO enrichment analysis indicated that this subgroup is associated with amyloid precursor protein metabolism, negative regulation of cell adhesion, and immune response regulation pathways. Amyloid precursor protein is associated with neurodegenerative diseases, and its metabolism and degradation may play an important role in the development of Subgroup III's disease. Genes such as ADAM17, ITM2B, PICALM, ROCK1, and RTN3 are involved in amyloid precursor protein metabolism and degradation. Additionally, the pathways related to negative regulation of cell adhesion and immune response are characteristic of Subgroup III and may be involved in cell-cell interactions and immune system function.

KEGG enrichment analysis for Subgroup I (genes from blue and turquoise modules) revealed significant enrichment in the Ribosome and Coronavirus disease - COVID-19 pathways, as well as metabolic pathways such as Phenylalanine metabolism, Legionellosis, Tyrosine metabolism, and biological processes like Drug metabolism - other enzymes, Spliceosome, and mRNA surveillance pathway. These results suggest that Subgroup I may be associated with viral infections and exhibit differences in cellular metabolism and gene expression regulation.

Subgroup II (genes from the pink module) KEGG enrichment analysis showed enrichment in pathways related to Glycosylphosphatidylinositol (GPI)-anchor biosynthesis, Autophagy - other, Thyroid cancer, Proteasome, and N-Glycan biosynthesis. These pathways are involved in cellular membrane structure, protein degradation, and cell signaling processes, suggesting that Subgroup II may play a critical role in the pathological development of RA.

Subgroup III (genes from the black, grey, and yellow modules) KEGG enrichment analysis revealed pathways associated with infection, such as Shigellosis, Salmonella infection, Pathogenic *Escherichia coli* infection, Yersinia infection, and pathways related to immune regulation and cell proliferation, such as Insulin resistance, Chronic myeloid leukemia, and TNF signaling pathway. These results suggest that Subgroup III may be closely related to infection and immune dysregulation and may play an important role in RA's inflammatory processes and immune dysfunction.

## 4 Discussion

In this study, we analyzed the gene expression profiles of RA cases using three independent transcriptome datasets and obtained a batch-corrected dataset to ensure uniformity. Through consensus analysis of 362 RA cases, we identified three highly concentrated subgroups. Subsequent correlation analysis involving clinical characteristics, relevant functional modules, and pathways demonstrated significant associations with these subgroups. Subgroup III exhibited elevated levels of ESR, MMP3, CRP, CDAI, and SDAI, indicating a more severe disease condition. Furthermore, the analysis revealed a close relationship between the classification of RA subgroups and clinical features, as well as specific functional modules or pathways.

The inspiration for this research stems from cancer subgrouping, which explores specific therapeutic targets through heterogeneous studies. Extensive research has been conducted on the transcriptional differences among these subgroups. For example, Etienne Becht et al. [19] reported that a favorable prognostic-enriched subgroup in colorectal cancer displayed overexpression of cytotoxic lymphocyte-specific genes, while an unfavorable prognostic subgroup expressed cell markers originating from lymphocytes and monocytes. Beyond cancer research, non-cancerous diseases, such as idiopathic inflammatory myopathy (Ingrid E Lundberg et al., 2018) [20], Alzheimer's disease (Sha Liu et al., 2022) [21], and heart failure (Virginia S Hahn et al., 2021) [22], have associated clinical phenotypes with subgroup analysis. These studies have advanced our understanding of the molecular mechanisms underlying subgroups and disease development, fostering personalized diagnostic and therapeutic approaches.

Similar to cancer, RA also exhibits clinical heterogeneity. In contrast to previous studies (Qi Cheng et al. [23], 2021; Yanzhi Ge et al. [24], 2021; Yukiko Maeda et al. [25], 2017), which merely compared RA gene expression profiles with those of normal control groups, our approach involved subgrouping RA cases, leading to the identification of distinct clinical features among different subgroups. For instance, Subgroup III showed a higher proportion of males compared to other groups and exhibited higher clinical feature indicators, such as ESR, MMP3, CRP, CDAI, and SDAI, suggesting a more severe disease condition.

In addition, we identified subgroup core genes through a Protein-Protein Interaction (PPI) network analysis. Core genes within Subgroup I play crucial roles in encoding ribosomal proteins essential for protein synthesis. For instance, genes such as RPS27A, RPS5, RPS9, RPS16, and RPS15A encode ribosomal small subunit proteins, crucial for constructing the small subunit of the ribosome, thereby facilitating protein synthesis [[26], [27], [28]]. Similarly, genes such as RPL3, RPL26L1, RPL6, and RPL39 encode ribosomal large subunit proteins, pivotal for constructing the ribosome's large subunit and are indispensable components of protein synthesis [[29], [30], [31], [32], [33]]. Furthermore, SNU13 encodes a cofactor crucial in ribosome biosynthesis, contributing to the regulation of ribosome assembly and maturation processes [34,35].

Genes within Subgroup II are involved in various biological processes, including protein synthesis, immune response, cell signaling, metabolism, and gene regulation. For example, GATA1 can lead to the upregulation of Cellular Communication Network Factor 6 (CCN6), which drives the pathogenesis of rheumatoid arthritis by altering the proliferative and angiogenic activity of synovial fibroblasts [36]. The encoded perforin 1 (PRF1), a protein involved in regulating cell-mediated cytotoxicity, affects the prognosis of rheumatoid arthritis and ankylosing spondylitis by modulating the dual cytotoxicity and regulatory properties of CD8^+^ T cells [37,38]. TNF receptor-associated factor 2 (TRAF2) influences the progression of RA by participating in cell survival, inflammatory response, and apoptotic signaling [39,40]. Furthermore, ETS1, a member of the ETS family of transcription factors, regulates the expression of numerous genes involved in cell differentiation and proliferation, and has been identified as a susceptibility locus for various autoimmune and inflammatory diseases [41]. Genes such as KARS1, HBQ1, PSMF1, SLC25A39, and ATIC participate in protein biosynthesis processes, mitigate reactive oxygen species levels, and promote cellular proliferation [[42], [43], [44], [45], [46], [47], [48], [49]].

Genes within Subgroup III are involved in various biological processes including immune regulation, cell signaling, inflammation regulation, cell proliferation, and apoptosis. For example, interleukin-1β (IL1B) is produced and secreted by various cell types, especially cells of the innate immune system (e.g., monocytes and macrophages), exacerbating damage during chronic disease and acute tissue injury, and playing a critical role in the host's defense response to infection and injury [50,51]. Signal transducer and activator of transcription 3 (STAT3) plays a critical role and has been demonstrated to be important in rheumatoid arthritis [[52], [53], [54]]. Furthermore, genes such as interleukin-1α (IL1A), matrix metalloproteinase 9 (MMP9), and growth factor receptor-binding protein 2 (GRB2) participate in regulating inflammatory responses and cell signaling [[55], [56], [57], [58], [59]]. A recent study on genetically informative alleles has found that TGFB1 predicts active disease in different subgroups of RA patients, suggesting an association between TGFB1 and moderate/severe active disease in RA, independent of autoantibody positivity [60], aligning with our conclusion that subgroup III may indicate more severe disease. A study of clinical specimens from RA revealed that Toll-like receptor 8 (TLR8) is highly expressed in synovial tissue, forming an extracellular trap for neutrophils [61]. PTEN, CCAAT enhancer binding protein beta (CEBPB), and suppressor of cytokine signal transduction protein 3 (SOCS3) are also implicated in the pathogenesis of rheumatoid arthritis through the regulation of cell proliferation, apoptosis, and immune cell function [[62], [63], [64], [65], [66]].

### 4.1 Biological functions of subgroups

#### 4.1.1 Subgroup I

Subgroup I is primarily associated with cytoplasmic translation, purine-containing compound biosynthesis, as well as AMP metabolism and biosynthetic pathways. These findings suggest significant regulation in protein synthesis and cellular metabolism within Subgroup I. GO enrichment analysis further revealed upregulation of genes related to purine metabolism and AMP synthesis, which may play critical roles in the metabolic state and inflammatory processes within Subgroup I. Moreover, in the KEGG enrichment analysis, Subgroup I was prominently enriched in the Ribosome and Coronavirus disease - COVID-19 pathways, along with multiple metabolic pathways and biological processes. These enriched pathways underscore the differences in virus infection, cellular metabolism, and gene expression regulation within Subgroup I. The enrichment of Ribosome pathway indicates a substantial activity in protein synthesis within this subgroup, which aligns with the results of the GO enrichment analysis related to cytoplasmic translation. These findings further emphasize the differences in protein synthesis, cellular metabolism, and gene regulation within Subgroup I, potentially related to virus infection. These results pique our interest in exploring the association between Subgroup I patients and virus infection, cellular metabolism, protein synthesis, and inflammatory responses.

The “bystander activation” theory refers to nonspecific and excessive reactive antiviral immune responses [67]. Virus infections can alter the clinical status of autoimmune diseases, such as enterovirus infections, including Coxsackievirus B (CVB) and rotavirus, as well as influenza A virus (IAV) and herpes viruses, which may contribute to RA pathogenesis [68]. Our findings strongly associate Subgroup I with virus infection, confirming the viral hypothesis in the pathogenesis of RA.

Subgroup I also demonstrated associations with cellular metabolism, protein synthesis, and inflammatory responses. In immune-mediated inflammatory diseases (IMID), alterations in local metabolism and the pathological effects of local hypoxia increase the biological energy and biosynthetic demands of immune and stromal cells [[69], [70], [71]].

#### 4.1.2 Subgroup II

The analysis of Subgroup II revealed involvement in mitochondrial and organelle autophagy, protein localization, and organelle disassembly pathways. This suggests potential abnormalities in intracellular metabolism and stress responses within Subgroup II. KEGG enrichment analysis also showed substantial enrichment of pathways related to cell membrane structure, protein degradation, and cell signaling, which may play important roles in the localization and maintenance of intracellular proteins and organelle structures during RA pathogenesis.

The specificity of genes in Subgroup II revealed heterogeneity in mitochondrial autophagy processes. Due to changes in mitochondrial autophagy function, the stability of organelles is affected, and damaged mitochondria are degraded through the autophagy-lysosome pathway [72].

There is evidence suggesting that autophagy plays a protective role in inflammatory diseases. For instance, impaired autophagy leads to increased susceptibility to intestinal inflammation [73], and reduced autophagic activity is associated with reduced bone mass in osteoporosis, while autophagy activation improves the symptoms [[74], [75], [76]].

#### 4.1.3 Subgroup III

Subgroup III is considered the most dangerous subgroup, involving amyloid precursor protein metabolism, negative regulation of cell adhesion, and immune response regulation pathways. GO enrichment analysis revealed upregulation of genes associated with neurodegenerative diseases, which may be related to abnormalities in neurological function and protein metabolism within Subgroup III. Additionally, Subgroup III is enriched in infection-related pathways and multiple immune regulatory pathways, further highlighting its association with infection and immune dysregulation.

The pathological physiology of Subgroup III can be described as abnormal amyloid precursor protein activation, promoting inflammatory responses, and cell proliferation. Previous studies [77] have found a positive correlation between amyloid precursor protein and CRP, consistent with the results of our correlation analysis.

Amyloid protein deposition is a chronic inflammatory disease that can induce the synthesis and secretion of various pro-inflammatory cytokines, including TNFα (activated by Subgroup III-specific gene ADAM17), IL-6, and IL-1β [[78], [79], [80]]. These factors may be responsible for the highest levels of inflammatory factors observed in Subgroup III, such as MMP3.

Overall, RA Subgroups I, II, and III exhibit distinct biological functions and enriched pathways in our bioinformatics analysis. These findings provide valuable clues for further exploring RA pathogenesis, personalized therapies, and the development of new therapeutic targets. However, to better understand these discoveries, further experimental validation and clinical research are needed to confirm their biological significance and potential mechanisms of action.

## 5 Limitations and Prospects

Limitations: Although this study conducted comprehensive analyses using three independent transcriptome datasets and successfully eliminated batch effects to obtain a uniformly batch-corrected dataset, there are still some limitations that warrant attention. Firstly, the relatively small sample size in this study may have restricted a comprehensive understanding of RA subgroups. Increasing the sample size could potentially aid in more accurately revealing the characteristics and functions of RA subgroups. Secondly, the definition and classification of subgroups in this research relied solely on transcriptome data without validation through other clinical features or biological markers. Incorporating more clinical information and biological indicators into the analysis might enhance the accuracy and clinical utility of the subgroups. Additionally, this study did not involve long-term follow-up data and clinical outcomes among the subgroups, resulting in a lack of information on treatment responses and prognoses among different subgroups, which limits a deeper understanding of the subgroups' significant roles in disease progression.

Prospects: Despite the aforementioned limitations, this study provides crucial insights and directions for the investigation and personalized treatment of RA subgroups. By subgrouping RA patients, we can better comprehend the heterogeneity and pathogenesis of RA. This provides robust evidence for identifying therapeutic targets specific to certain subgroups and developing personalized treatment strategies. By further integrating clinical features and biological markers, we can more precisely identify different subgroups, leading to more targeted treatment approaches. Additionally, exploring transcriptional and functional differences among subgroups may reveal distinct mechanisms involved in inflammation and immune regulation, thus offering comprehensive information for disease treatment and prognosis assessment.

## 6 Conclusions

Precision treatment of RA has long been a global challenge. Through bioinformatics analysis of molecular subgroups among RA patients, we have identified three potential subgroups, each exhibiting distinct enrichment features in biological pathways. These findings are expected to provide essential evidence for further investigating the pathogenesis, disease classification, and personalized treatment of RA, facilitating the development of more targeted therapeutic strategies and offering more accurate information for personalized treatment and prognosis assessment. Overall, this study presents new insights for gaining a deeper understanding of the molecular mechanisms of RA, identifying potential therapeutic targets, and developing personalized treatment approaches.

## Funding

This work was financially supported by the Guangdong Provincial Medical Science Foundation (Grant numbers A2020103, 20201A011019) to Qq.M.; the major project of Bijie Bureau of Science and Technology (Grant numbers [2022]-1) to Qq.M.; the Science and Technology of Program of Guangzhou (Grant numbers 202102080344) to Qq.M.

## Data availability statement

The data that support the findings of this study are available from the corresponding author upon request.

## CRediT authorship contribution statement

**Yangyang Xu:** Writing – review & editing, Writing – original draft, Software, Methodology, Conceptualization. **Zhenyu Yang:** Writing – review & editing, Methodology, Conceptualization. **Tengyan Wang:** Validation, Software. **Liqiong Hu:** Writing – review & editing, Validation. **Songsong Jiao:** Validation, Software, Formal analysis. **Jiangfei Zhou:** Formal analysis. **Tianming Dai:** Methodology, Data curation. **Zhencheng Feng:** Validation. **Siming Li:** Supervision, Conceptualization. **Qinqqi Meng:** Supervision, Conceptualization.

## Declaration of competing interest

The authors declare that they have no known competing financial interests or personal relationships that could have appeared to influence the work reported in this paper.
